# Epigenetics in Turner syndrome

**DOI:** 10.1186/s13148-018-0477-0

**Published:** 2018-04-06

**Authors:** Francisco Álvarez-Nava, Roberto Lanes

**Affiliations:** 1 Biological Sciences School, Faculty of Biological Sciences, Central University of Ecuador, Quito, Ecuador; 2 Pediatric Endocrine Unit, Hospital de Clínicas Caracas, Caracas, Venezuela

**Keywords:** Aneuploidy, Chromatin, DNA methylation, Embryonic stem cells, Epigenetics, Gene expression, Mouse models, Turner syndrome

## Abstract

**Background:**

Monosomy of the X chromosome is the most frequent genetic abnormality in human as it is present in approximately 2% of all conceptions, although 99% of these embryos are spontaneously miscarried. In postnatal life, clinical features of Turner syndrome may include typical dysmorphic stigmata, short stature, sexual infantilism, and renal, cardiac, skeletal, endocrine and metabolic abnormalities.

**Main text:**

Turner syndrome is due to a partial or total loss of the second sexual chromosome, resulting in the development of highly variable clinical features. This phenotype may not merely be due to genomic imbalance from deleted genes but may also result from additive influences on associated genes within a given gene network, with an altered regulation of gene expression triggered by the absence of the second sex chromosome. Current studies in human and mouse models have demonstrated that this chromosomal abnormality leads to epigenetic changes, including differential DNA methylation in specific groups of downstream target genes in pathways associated with several clinical and metabolic features, mostly on autosomal chromosomes. In this article, we begin exploring the potential involvement of both genetic and epigenetic factors in the origin of X chromosome monosomy. We review the dispute between the meiotic and post-zygotic origins of 45,X monosomy, by mainly analyzing the findings from several studies that compare gene expression of the 45,X monosomy to their euploid and/or 47,XXX trisomic cell counterparts on peripheral blood mononuclear cells, amniotic fluid, human fibroblast cells, and induced pluripotent human cell lines. From these studies, a profile of epigenetic changes seems to emerge in response to chromosomal imbalance. An interesting finding of all these studies is that methylation-based and expression-based pathway analyses are complementary, rather than overlapping, and are correlated with the clinical picture displayed by TS subjects.

**Conclusions:**

The clarification of these possible causal pathways may have future implications in increasing the life expectancy of these patients and may provide informative targets for early pharmaceutical intervention.

## Background

Over 80 years ago, Turner [1] first described a clinical picture that would subsequently carry his name. Since then, a great wealth of knowledge has been accumulated regarding the monosomy of sex chromosomes. It was Ford [2] who settled the cytogenetic basis and Ferguson-Smith who proposed the existence of genes in the short arms of both sex chromosomes that would determine the Turner syndrome (TS) phenotype [3]. From there on, the cytogenetic findings in patients with this disorder have been linked to molecular sources allowing us to better understand its pathogenesis. The girls with TS have meanwhile grown up, and in recent years, a new phenotype for this old condition, related to its morbidity and mortality, has been outlined. Although researchers have tried to explain the clinical findings in TS based on cytogenetic and gene changes, everything seems to indicate that other causes for this disorder, beyond the nucleotide sequence of a gene or the global impact of the chromosomal imbalance, exist. These modifications need to be explored, beginning with the primary etiology that causes the inadequate segregation of the sex chromosomes, up to the metabolic disorders that appear in adult life and which tend to diminish the life expectancy of women with TS. Similarly, from this perspective, short stature, gonadal dysgenesis, and congenital heart disease, as well as intrauterine growth retardation (IUGR), epidemiologically related to metabolic problems in other disorders, must be examined.

Evidence collected in recent years does not seem to indicate only one proposed mechanism to explain the clinical findings and the associated complications in TS, but instead the concurrence of several of them. In addition, there does not appear to be an association between the primary cause that determines the aneuploidy of the sex chromosomes and the clinical findings. A unifying hypothesis to help explain the findings in the embryonic, fetal, neonatal, and adolescent periods, as well as in adulthood, and the pathogenesis of this disorder, relates to changes in DNA conformation and its assembly without modifying its nucleotide sequence. This review describes the latest known facts on the role of epigenetics in the etiopathogenesis of TS. We are now in a stage in which TS transcends its clinical, cytogenetic, and gene aspects into the epigenetic landscape.

## General considerations

Although it has been known since 1959 [2] that TS is caused by a complete or partial deletion of the second sex chromosome, the mechanisms by which monosomy of the X chromosome disrupts development is still not well understood. Several deleted genes from the X chromosome are expected to largely affect various tissues, organs, and systems during embryonic development, growth, and adult life. This hypothesis is known as the “Gene Dosage Effect,” which explains the cause of TS based on the absence of a limited number of dosage sensitive genes localized on the Xp chromosome [3–5]. In other words, this hypothesis states that developmental, clinical, or metabolic features of TS can be mapped to specific regions of sex chromosomes. Thus, based on this hypothesis, the TS phenotype is a direct consequence of the cumulative effect of the absence of individual loci (haploinsufficiency) located on the Xp chromosome [6]. The Gene Dosage Effect hypothesis is mainly supported by rare cases of partial Xp deletion, linking TS phenotypes to a small number of loci in specific segments of the Xp chromosome. However, genes do not operate as independent units in the genome but are rather embedded in temporally and spatially highly coordinated regulatory networks. Thus, the “Reductionist” view contrasts with the “Organicistic” view or “Amplified DevelopmentalInstability” hypothesis which is basically based on Waddington’s view. It states that the pathogenesis of TS is strictly connected to the presence of a monosomy of the second sex chromosome that profoundly disturbs genomic homeostasis, i.e., the TS phenotype is the result of global genomic imbalance, rather than the addition of the effects of individual loci [7, 8]. Nevertheless, recent research inclines us to support a synthesis of these two views, which indicates that with a deleted gene, the individual effect is modest and one could therefore only explain the TS phenotype by combination with other loci, based on the specificity of effects and interactions of these genes [9]. Thus, the TS phenotype may not merely be due to genomic imbalance from deleted genes linked to the second sex chromosome but may also be due to additive influences on associated genes within a given gene network with an altered regulation of gene expression triggered by epigenetic factors.

Understanding the role of genetics in establishing epigenetic patterns has become a high priority in the postgenomic era. Epigenetic mechanisms include all the processes involved in creating instructions for a cell to function efficiently. To carry out this action, epigenetic machineries perform an important role in controlling gene expression and, thus, regulating normal and abnormal cellular processes associated with diseases. Pathological conditions occur when epigenetics become decontrolled. Most evidence suggests that epigenetic modifications are carried out by four mechanisms: (1) DNA methylation of CpG sites in the promoter region of the gene, (2) posttranslational covalent modifications of histones (histones code) and the use of variant histone proteins, (3) remodeling of nucleosomes and/or reorganization of chromatin on a larger scale, and (4) regulation of gene expression by small, noncoding RNA molecules (microRNA, miRNA). These modifications are reversible, changing the chromatin configuration (open or closed), activating or silencing genes, and do not occur in isolation, i.e., the overall effect is obtained by the combination, type, site, and extent of modifications. Increasing evidence demonstrates that epigenetics has a powerful impact on normal cognition, cardiovascular development, growth, and lipid and glucose metabolism [10–12]. However, epigenetic effects have been poorly investigated in TS as, up to now, most of the TS research has focused on clinical, chromosomal, and genetic abnormalities (Reductionist view).

Hypothetically, the haploinsufficiency of loci on the Xp chromosome results in a 0.5-fold diminished gene expression. However, transcriptome analyses in different human tissues and in XO mouse models did not reveal a direct correlation between genomic imbalance and gene expression levels [13–17]. Significantly, changes in gene expression have been also detected in loci that are not on the X chromosome and diverge between cell and tissue types [13–16, 18]. It is important to emphasize that many genes display broad expression variation and, even for genes that are deregulated in TS, there is an extensive overlap of expression levels between 46,XX and 45,X cells [13, 19]. Thus, it is reasonable to assume that this variation in gene expression significantly contributes to the phenotypic variation seen in TS individuals. In addition, copy number variations might also explain such variable expressivity in TS [20]. Specific clinical features exhibit a broad spectrum in TS subjects, even in subjects with 45,X karyotype. Thus, copy number variations might help explain such variable expressivity and be implicated in neurodevelopmental disorders, thoracic aortic aneurysms, and dissections in TS [20]. However, it seems premature to conclude whether copy number variations are an influencing factor in the TS phenotype. On the other hand, the functional consequences of epigenetic changes, which occur at a higher rate than DNA sequence changes, are likely underestimated in this chromosomal disorder. While haploinsufficiency of genes on the X chromosome has been the focus of recent research, underlying epigenetic mechanisms have been poorly studied in TS. Nevertheless, it is has become clear that epigenetic processes are altered in TS [13–19], so that by modulating gene expression, epigenetics could play a crucial role in altered growth and in the development of abnormalities of lipid and glucose metabolism associated with TS. While this has not been clearly demonstrated so far, it could assist in deciphering the epigenetic regulation of human IGF1 gene expression, which would be useful in the diagnosis of growth and bone metabolism alterations and for the management and rational use of rhGH therapy. Analyzing the status of DNA methylation in the PPARGC1A promoter and the biogenesis of mitochondria and their relevance on the development of metabolic abnormalities may provide new biomarkers that could predict cardiometabolic risk in women with TS (see the “Summary and future perspectives” section).

Due to recent advances in high throughput screening of both mRNA expression and DNA methylation as a resource to study epigenetic mechanisms, an increasing wealth of knowledge regarding gene expression has been emerging. These recent advances have allowed us to understand the essential connection between gene clusters in effecting specific pathways. This review begins by exploring the potential involvement of both genetic and epigenetic factors in the origin of X chromosome monosomy. Several in vivo and in vitro studies have examined the effects of epigenetic alterations on the remodeling of chromatin, which disturb the ability of the chromosomes to align, attach to mitotic spindle fibers, and/or separate. These studies have led to the identification of pathways affected in non-disjunction chromosomal conditions, such as Immunodeficiency, Centromeric instability and Facial anomalies (ICF) and Mosaic Variegated Aneuploidy syndromes. We also review the dispute between the meiotic and post-zygotic origins of the 45,X monosomy. The meiotic epigenetic landscape of sex chromosomes is faced with the chromosomal instability of blastomeres in early embryonic development, mediated by post-zygotic epigenetic reprogramming in order to explain the high frequency of X-monosomy in human conception. Subsequently, we focus on the phenotypical consequences of epigenetic modifications in 45,X monosomy, as a profile of epigenetic changes that seem to emerge in response to chromosomal imbalance caused by the absence of the second sex chromosome. This epigenetic pattern may contribute to both the clinical picture and the phenotypic variation present in TS patients. We will also discuss the debate that exists between Reductionists and Organicistic, through which gene expression regulated by epigenetic mechanisms allows us to establish a synthetic view of the clinical picture and the phenotypic variability of TS. Thus, we analyze the findings from several studies that compare gene expression of 45,X monosomy to their euploid and/or 47,XXX trisomic cell counterparts on peripheral blood mononuclear cells, amniotic fluid, human fibroblast cells, and induced pluripotent human cell lines. We also review studies that have provided evidence of an altered methylome in 45,X fibroblast cell lines, as well as in peripheral blood mononuclear cells. These in vitro results have been corroborated in vivo by analyzing the methylation profile in a well-characterized cohort of women with 45,X monosomy [13]. An interesting finding of all these studies is that methylation-based and expression-based pathway analyses are complementary, rather than overlapping, and are correlated with the clinical picture displayed by TS subjects. The clarification of these possible causal pathways may have future implications in increasing the life expectancy of these patients, as epigenetics could potentially pinpoint central avenues of research for the management and treatment of short stature, bone disorders, metabolism, and cardiovascular diseases in this condition. Epigenetic signatures such as histone modifications and DNA methylation are reversible, unlike the monosomy of the second sex chromosome, and, thus, offer an enormous potential in reducing the effects of the chromosomal imbalances seen in TS. Studies currently being carried out suggest that changes in DNA methylation in TS patients may influence lipid metabolism and mitochondrial biogenesis (unpublished data). These ongoing studies may provide informative targets for early pharmaceutical intervention, so as to ameliorate alterations that confer an increased risk of cardiovascular and cerebrovascular diseases to patients with TS. Lastly, we discuss how the haploinsufficiency of Xp chromosome genes may cause a number of epigenetic consequences that may affect the appearance of clinical features, associated complications, and phenotypic variability in TS.

## Epigenetic origin of the 45,X monosomy

It has been speculated that several chromosomal heteromorphisms in certain heterochromatic regions can directly affect chromosomal malsegregation, possibly by compromising the fidelity of the spindle attachments and replication/pairing processes [21–23]. Large blocks of heterochromatin between homologous chromosomes could lead to asynchronous replication, which, in turn, may contribute to misalignment and nondisjunction [24, 25]. Additionally, the role of heteromorphisms in chromosomal nondisjunction could be due to disturbances in recombination, which, in turn, could lead to aneuploidy [26, 27]. However, the influence of chromosomal heteromorphisms appears to be minimal or to act through complex mechanisms (see below), and its association with aneuploidy has not been consistently observed.

More than 100 genes could provoke defects in chromosomal segregation when they are mutated, which may lead to mitotic spindle alterations, centromere amplification, cell cycle checkpoint defects, non-separation of sister chromatids, and telomere [24]. However, current evidence points out that such mutations are uncommon in aneuploidy cells [28, 29]. Thus, chromosomal behavior may be affected by other mechanisms that do not include chromosomal heteromorphisms or gene mutations.

Epigenetic mechanisms may serve as a potential link for explaining the two hypotheses mentioned above regarding chromosomal malsegregation [25]. For example, epigenetic alterations in pericentromeric heterochromatin may lead to remodeling in chromatin conformation which may compromise the ability of the chromosomes to align, attach to mitotic spindle fibers, and/or separate [24, 30]. This latter hypothesis is based on analyses of hypomethylated cells obtained either from cells treated with DNA-methyltransferase inhibitors (5-azacytidine) or from cells from patients with ICF syndrome who have mutations in the *DNA methyltransferase 3b* gene (*DNMT3B*). This gene encodes a DNA methyltransferase which is thought to function in de novo methylation, rather than in maintenance methylation which is an epigenetic mark. These analyses showed delays in centromere separation that led to aneuploidies [24]. Thus, hypomethylation in whole chromosome (for example in an X chromosome) might cause chromosomal instability and large-scale chromosomal changes. Consequently, DNA methylation and histone modification have an impact on the correct chromosomal segregation. On the other hand, genes involved in spindle checkpoint control could not be mutated but are instead aberrantly silenced in aneuploidy cells by epigenetic signature [29]. Thus, epigenetic mechanisms can replace mutations as a way of silencing the spindle checkpoint gene. Therefore, abnormal chromosomal segregation can be originated by alterations in DNA methylation on spindle checkpoint genes.

Although the role of epigenetic changes on the initiation of malsegregation of sex chromosomes is still largely speculative, a profile of epigenetic changes seems to emerge in response to chromosomal imbalance caused by 45,X monosomy, which may not only contribute to the clinical picture present in TS patients but can also be associated to malsegregation of sex chromosomes. Thus, a genome-wide hypomethylation has been described in leukocytes from 45,X patients [13], which might reactivate cryptic site transcription start and cause changes in expression of isoform transcripts. Applying analysis of differential exon usage to the autosomes in 45,X cell lines, eight protein coding and two non-coding RNAs genes differentially spliced have been described [13]. Of these genes, the *BB1* gene is striking since it encodes a kinase involved in spindle checkpoint function and chromosome segregation [31]. The *BB1* gene has been localized to the kinetochore and plays a role in the inhibition of the anaphase-promoting complex/cyclosome (APC/C), delaying the onset of anaphase and ensuring proper chromosome segregation. Impaired spindle checkpoint function has been found in many forms of cancer cells [24] and might play a role in the Mosaic Variegated Aneuploidy syndrome. This raises the possibility of altered expression of *BB1* predisposing to chromosomal loss during mitosis and playing a role in the loss of X chromosome material in TS [32]. Obviously, it must be recognized that the differential exon usage of *BUB1B* may also be a consequence of the 45,X monosomy itself [13]. Further studies are required to discern the role of this gene in other sex chromosome aneuploidies.

Similarly, through studies on embryonic stem cells (ESCs), Robertson et al. and Zvetkova et al. have described a general DNA hypomethylation in XX ESCs that generate XO cells comparable to XY ESC [33, 34]. As DNA hypomethylation increases and XO cell generations are gradually acquired, the X-to-autosome ratio decreases similar to that observed in control XY cells, indicating a loss of one of the two X chromosomes during serial passaging [34]. Thus, this globally reduced methylation level is associated to X chromosome instability. Additionally, methylation of differentially methylated regions (DMR) can be restored, but it is coincident with complete loss of an X chromosome in ESCs. Observations indicate that DNA hypomethylation in XX ESCs is attributable to the presence of two (active) X chromosomes rather than to the absence of a Y chromosome [34]. Global DNA hypomethylation and complete or partial deletion of DNA sequences from one of the two X chromosomes are associated with reduced levels of the de novo DNA methyltransferases Dnmt3a and Dnmt3b. Therefore, it would seem that global DNA hypomethylation in XX ESC lines occurs gradually during the embryonic passage of these cell lines and is probably due to a reduced level of the de novo DNA methyltransferases Dnmt3a and Dnmt3b. DNA hypomethylation can be restored in the late phase of embryonic passage of these ESCs and is coincident with complete or partial loss of DNA sequences from one of the two X chromosomes [34]. These findings support the hypothesis of retention of methylation provides a selective pressure for deletion of all or part of one X chromosome. Thus, the selection against loss of methylation may provide the basis for X chromosome instability.

Given the limited evidence in humans, the findings described in the XO mouse models can only be extrapolated in a speculative way to the humans. Unlike their human counterparts, in which approximately 15% of the X-linked genes escape X inactivation [35], in the XO mice, only a few “escapees” [36–38] have been reported. This has been used as evidence demonstrating the mouse X chromosome is depleted of genes escaping X inactivation, via DNA methylation, which may explain why the XO mouse has a near normal phenotype [39]. However, although the DNA methylation pattern on the X chromosome observed in mice differs from humans, several similarities have been reported in early embryonic development in both species. (1) The establishment of stable XX ES cell lines from both mouse and human blastocysts is relatively problematic owing to frequent loss of one of the two X chromosomes [34, 40]. (2) DNA hypomethylation is globally detected in both murine and human cell lines [13, 33, 34, 41, 42]. (3) This global hypomethylation can be a causal factor of the instability of the X chromosome which would condition early loss of the second sex chromosome in both systems. (4) The mechanisms of DNA methylation in eutherian mammals have been evolutionarily conserved. DNA methylation plays an important role in the regulation of gene expression [43], genomic imprinting [44], and X chromosome inactivation [45]. In eutherian mammals, two de novo DNA methyltransferases, Dnmt3a and Dnmt3b, have been shown to be essential for early embryonic development [46].

Human DNMT3A and DNMT3B share 98 and 94% of their amino acid sequence identity with the mouse Dnmt3a and Dnmt3b [47], which may indicate that their expression is regulated in a similar way and that alterations in their functions could originate a similar phenotype. For example, studies of *Dnmt3b* null and ICF mutant mice have shown that *Dnmt3b* is essential for mouse embryonic development and that the ICF mice exhibit phenotypes that resemble some of the symptoms of the human ICF syndrome [48]. All of this evidence suggests that it is feasible that the same events that occur in the embryonic passage of these cell lines in the murine model could be similar to those occurring in the human system.

## Meiotic versus post-zygotic origin of 45,X monosomy

Meiosis is the cellular division that underlies haploid gamete development. To achieve the reduction in diploid genetic complement needed for gamete formation, one round of DNA replication (S phase) is followed by two rounds of chromosome segregation (meiosis I (MI) and II (MII)). Although the role of the meiotic process is to generate gametes having half the genetic complement of the germ cells, during meiosis, genetic recombination occurs so as to increase genetic diversity. Genetic recombination involves the pairing and synapsis of homologous chromosomes, followed by genetic information transfer (crossover) between regions of homologous sequences of non-sister chromatids of homologous chromosomes. Additionally, genetic recombination also plays a mechanical role in the segregation of homologous chromosomes when they separate at MI. This chromosomal segregation at MI is ensured by the presence of physical connection between homologous chromosomes (chiasmata). It has been suggested that reduced homologous regions between X and Y chromosomes in male meiosis is particularly susceptible to chromosomal nondisjunction [49]. Thus, due to the heterologous nature of sex chromosomes, it is not surprising that the nondisjunction rate for sex chromosomes is higher than that for autosomes in male human meiosis [50]. Single sperm typing demonstrates that reduced recombination is associated with the production of aneuploid 24,XY human sperm [51]. Therefore, although autosomal aneuploid conditions are predominantly maternal in origin [52], sex chromosomal aneuploidies occur frequently because of nondisjunction in the meiosis male [53, 54]. In support of this assertion, several studies have shown that two thirds of 45,X monosomy individuals retain the maternal X chromosome [55–60]. In addition, an increased aneuploidy frequency for sex chromosomes relative to that of the autosomes in both human sperm karyotypes [61, 62] and FISH analysis of sperm [49, 63] supports this concept.

A characteristic feature of MI is genetic recombination which is initiated by the formation and repair of induced double-strand breaks (DSBs). Defects in genetic recombination disrupt DSB repair and result in checkpoint activation. In turn, checkpoint activation leads to either apoptosis or the formation of aneuploid gametes. Thus, it is critical that germ cells monitor these events and ensure they occur properly. However, heteromorphic chromosomes of sexually reproducing organisms (heterogametic sex) are either partially (e.g., XY in human, other mammalian, and Drosophila males) or completely hemizygous (e.g., X0 in a number of insects and worms), representing a special challenge to the repair of DSBs and, moreover, in evading checkpoint activation. Consequently, the underlying meiotic program of sex chromosomes must be epigenetically modified to promote adequate sex chromosome segregation during meiosis. Therefore, DSB repair and checkpoint suppression must occur in the context of a specialized chromatin structure found only on the X chromosome of males [64]. Specific silencing epigenetic marks on sex chromosomes alter interactions with DSB repair and checkpoint machinery to ensure accurate transmission of the male genome through meiosis, so as to achieve suitable sex chromosome segregation. Thus, during mammalian male meiosis, the heteromorphic sex chromosomes undergo a silencing process called Meiotic Sex Chromosome Inactivation (MSCI) [65, 66] which results in acquisition of repressive chromatin and transcriptional silencing. In *Caenorhabditis elegans*, MSCI is mediated by MET-2 methyltransferase deposition of histone H3 lysine 9 di-methylation (H3K9me2). MSCI is important for shielding the hemizygous X chromosome linked gene expression from checkpoint machinery [67]. It is essential for preventing the expression of a small number of deleterious Y chromosome-linked genes during male meiosis in mice, which would result in pachytene arrest and elevated germline apoptosis [68, 69].

Thus, alterations in the pathways that mediate DSB repair on the hemizygous regions of sex chromosomes in male meiosis could lead to sex chromosome monosomic gametes. Variations in the recruitment of DSB processing factors and checkpoint proteins to DSBs in germ lines with altered chromatin could modify the specific histone code on DSB repair and checkpoint silencing. Also, analyses of germline chromatin epigenetic signatures in *Caenorhabditis* species indicate that either H3K9me2 or H3K9me3 can be enriched on sex chromosomes and mediate transcriptional silencing [67]. Modifications of these epigenetic marks could lead to checkpoint activation and consequently to the formation of nullisomic gametes for sex chromosomes. However, further studies are necessary so as to understand the increased frequency of sex chromosome aneuploidy associated with human meiosis resulting in developmental disorders including Turner and Klinefelter syndromes.

On the other hand, although chromosomal instability is a well-known feature in cancer cells, it also is a prominent trait in early embryonic development. Early embryonic cell division is distinguished by the suppression of the cell cycle checkpoints [70], with epigenetic reprogramming of the genome and total demethylation of DNA sequences to exclude imprinted loci (totipotent stem cells). Consequently, chromosomal instability in blastomeres occurs. Therefore, abnormal post-zygotic epigenetic reprogramming can cause catastrophic consequences. Molecular cytogenetic techniques for pre-implantation genetic diagnosis of individual blastomeres have demonstrated that 15–85% of early embryos have numerical chromosomal abnormalities [71–74]. This chromosomal instability involves monosomy–trisomy for whole chromosomes, parental disomies, and complex patterns of segmental deletions, duplication, and amplification that are reciprocal in sister blastomeres, implying the occurrence of breakage–fusion–bridge cycles [72]. These findings correspond on the one hand with the complex structural chromosomal aberrations observed in individual with birth defects [74], and on the other hand, they also explain the high incidence of aneuploidy embryonic losses (failure in the implantation process). Thus, monosomy of the sex chromosomes could be a relatively common event and explain the high lethality observed in 45,X embryos.

Although meiotic nondisjunction is assumed to be the principal mechanism in explaining the aneuploidies of sex chromosomes, several lines of evidence suggest that 45,X monosomy may have a post-zygotic origin in accordance with the early embryonic chromosomal instability mentioned above. First, the incidence of 45,X karyotypes in early embryos is notably higher than that inferred of nullisomic gametes for a sexual chromosome [75, 76]. Second, in contrast to the majority of constitutional autosome aneuploidies, the 45,X conceptuses that retained paternal X chromosome are not associated to maternal age, i.e., are not caused by a maternal meiosis I error [77]. Thirdly, a very large proportion of 45,X embryos acquired their 45,X cell line after in vitro fertilization [78, 79]. Lastly, human embryonic stem cell lines loose one sex chromosome during in vitro passage [40]. Thus, the most likely scenario seems to be that of 45,X monosomy arising post-zygotically through a mitotic error.

One hypothesis to explain the survival of the post-zygotic 45,X cell line in contrast to the normal euploid cell line (46, XX or 46 XY) is that epigenomic deregulation could promote rapid survival selection of 45,X cells. Thus, the loss of a second sexual chromosome may introduce a global epigenetic effect as a cellular response to overcome the detrimental effects of monosomic cells. This may indirectly facilitate the process of adaptive cells by producing a fitness advantage only after a secondary and required genetic event has occurred [77].

## Phenotypical consequences of the epigenetic modifications in 45,X monosomy

The clinical consequences logic attributed to 45,X are direct dosage effects of genes on the sexual chromosomes, i.e., TS is due to complete or partial loss of DNA sequences in the second sex chromosome which provokes haploinsufficiency of genes that are normally biallelically expressed from both sex chromosomes and escape from X chromosome inactivation. In humans, ~ 15% of X-linked genes escape X-inactivation [80, 81] so that only a small number of genes are predicted to contribute to dosage imbalances in 45,X monosomy. A large effort has gone into attempting to identify genomic regions on sex chromosomes that contain the critical genes associated with the TS phenotype. Cytogenetic characterization of structurally abnormal X and Y chromosomes has provided support for the hypothesis of distinct TS loci and a unique candidate gene probably does not exist. Consequently, certain clinical features in TS have been mapped to specific areas of the sex chromosomes: short stature to the X and Y tip of the short arm and ovarian function to both the short and long arms, i.e., different clinical features may be due to different genes. Although several candidate genes have been proposed to explain what causes the TS phenotype, the only gene that has been proven to be associated with clinical features of TS (skeletal anomalies and short stature) is the SHOX gene (short stature homeobox-containing gene, NM000451) [82, 83]. Therefore, the prevailing hypothesis of a gene dosage imbalance following loss of one sex chromosome material is questioned. Thus, other hypotheses to explain both the clinical features and the variability of expression in TS have been proposed.

The absence of the second sexual chromosome in TS has led authors to speculate that there may be genes present on the X chromosome which are expressed differently depending upon whether they are maternally or paternally inherited [55, 56, 58–60, 79, 84–88]. In other words, a 45,X TS patient has an increased likelihood of certain features depending on whether her X chromosome was inherited from her mother or father. It seemed based on a number of original studies that patients with TS that inherited their only X chromosome from their mother had an increased incidence of cognitive function disorders, visceral adiposity, and atherogenic lipid profiles, similar to that of men [55, 59, 84, 89], which led to the hypothesis of X-linked imprinted genes. However, no human genomic imprinted genes on the X chromosomes have been identified, and no significant skewed parental origin effect of the clinical features of TS patients has been recognized, with the exception of those exhibiting sexual dimorphism. In addition, other studies have provided conflicting results in relation to X-linked parental effects. Furthermore, the parental origin of the retained X chromosome may be a confounder factor of clinically important parameters. Therefore, from a clinical perspective, a genetic work-up to detect the parental origin of the remaining X is currently not indicated in routine care of women with TS [90]. Alternatively, transcriptome studies have implicated several genes with altered expression involved in pathways controlled through epigenetic regulation [13–18]. They may contribute directly to known pathological mechanisms identified on prior gene expression profiling in genes associated to autoimmune diseases; urogenital congenital malformations; obesity and metabolic disorders, including type 2 diabetes mellitus (T2DM) and metabolic syndrome; fetal development and embryonic lethality; sensorineural hearing loss; and aneuploidies [13, 15, 17]. The TS phenotype may therefore likely arise from an abnormal connection of various genetic and epigenetic factors whose primary source is the monosomy of the second sex chromosome. On the other hand, the effects of genetics on epigenetic modeling can be clustered into those that occur in *cis*, such as short-range effects of SNPs and haplotypes, and those that occur in *trans*, including the effects of 45,X monosomy on chromatin states [20]. In TS, another important class of *trans*-acting genetic effects could be due to deleted second sex chromosome genes which code epigenetic “reader” and “writer” enzymes. Increasing evidence suggests that TS features could be caused by altered regulation and complex interrelations of many genes both on and outside both sex chromosomes [13, 14, 16–19].

Several studies have compared global gene expression of 45,X monosomy on peripheral blood mononuclear cells [84, 91], amniotic fluid [92], human fibroblast cells [15, 19] and induced pluripotent human cell lines [16] to their euploid and/or 47,XXX trisomic cell counterparts. Not surprisingly, 45,X cells display a differential global gene expression pattern separable from euploid and 47,XXX cells. However, it should be noted that the most differentially expressed genes were not located on the retained X chromosome. Moreover, thousands of autosomal genes are differentially expressed between the sexes in several somatic tissues, with 14% (brain) to 70% (liver) of active genes affected [93, 94].

To analyze the functional interpretation of large lists of genes derived from microarray studies, *Database for Annotation*, *Visualization and Integrated Discovery* (DAVID) has been employed as an online bioinformatic resource. By using a novel cDNA based microarray approach, differential methylation in human X chromosome in 45,X, 46,XX, and 47,XXX cells has been demonstrated [14–16]. These studies utilized low-resolution DNA methylation analyses and have provided evidence of an altered methylome in 45,X fibroblast cell lines [14, 15, 19], as well as in peripheral blood mononuclear cells [17, 84, 91, 95]. In a study, DAVID analysis revealed a differential methylation profile of genes associated with nuclear chromosome condensation and chromatin remodeling complex involving histone modification [19]. When the expression profile of 45,X and 46,XX were compared, the “skeletal system,” “gonadal development and function,” “glucose metabolism,” and “epigenetic regulation” pathways were found to be differentially expressed in 45,X cells. Interestingly, this methylation profile correlates with the clinical picture that is displayed by TS subjects [19]. However, these studies did not examine a well-characterized cohort of women with 45,X monosomy. In this context, the leukocyte DNA-methylation profile was recently investigated when 45,X, 46,XX, and 46,XY subjects were compared by using the 450K-Illumina Infinium assay [13]. Genome-wide X chromosome RNA expression, autosomal DNA-methylation, and X chromosome methylation profiles clearly discriminated TS subjects from controls. This study demonstrated genome-wide hypomethylation, with the most DMRs showing a medium level of methylation. This hypomethylation status was extended to repetitive elements. Interestingly, novel genes including several escape genes, X-Y homologous gene pairs, and pseudoautosomal genes were identified. These genes could be related to comorbidities in TS such as autoimmune diseases, urinary congenital malformations, premature ovarian failure, and aortic aneurism formation [13]. Methylation of CpG islands in gene promoter regions during development, growth, or disease processes is associated with posttranslational histone modifications that lead to a locally condensed inactive chromatin structure and gene silencing. Genome-wide methylation studies have identified this epigenetic signature in 45,X cells in several tissues, including leukocytes, skin fibroblasts, buccal cells, liver, and placenta, adding another layer of complexity to the highly variable clinical features of TS. Interestingly, X chromosome carries several genes that are key players in epigenetic regulation.

**Fig. 1:**
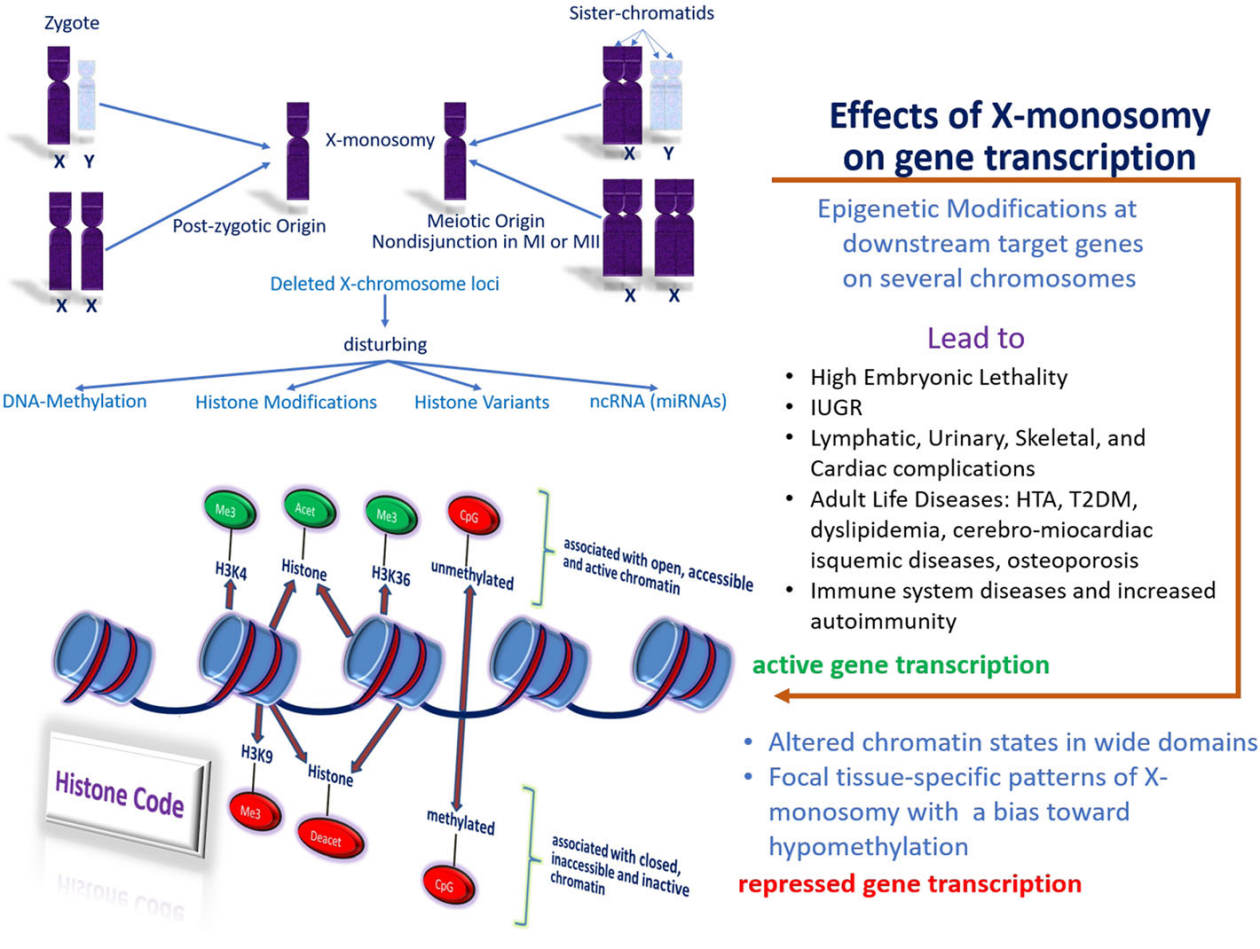
Effects of X chromosome monosomy on gene transcription. X chromosome monosomy and epigenetic modifications. DNA is packed (compacted) into chromatin (DNA associated with histone and non-histone proteins) whose packaging unit is the nucleosome: 147 base pairs of DNA wound around an eight-histone protein (octamer) core. Gene expression rests on the chromatin state: (a) an open, accessible, and active chromatin (euchromatin) is associated with gene expression due to the combination of an histone code (H4Kac, H3K4, H3K36) and un(hypo)methylated CpG islands, which allow access of the transcriptional machinery (RNA polymerase and associated factors), and (b) a closed, inaccessible, and inactive chromatin (facultative heterochromatin) is associated with gene silencing due to the combination of an histone code (histone deacetylation, H3K9me3, H3K27me3) and (hyper)methylated CpG islands, which prevent access of the transcriptional machinery. H4Kac: lysine acetylation at histone 4; H3K4: methylation of lysine 4 residues at histone 3; H3K36: methylation of lysine 36 residues at histone 3; H3K9me3: trimethylation of lysine 9 residues at histone 3; H3K27me3: trimethylation of lysine 27 residues at histone 3; CpG: un-, hypo-, hyper-methylated: addition or removal of methyl groups to the 5′-carbon of cytosine, especially on CpG dinucleotides enriched in small regions of DNA (< 500 bp); MI or MII: meiosis I or meiosis II; ncRNA: noncoding RNA; miRNA: micro-noncoding RNA; IUGR: intrauterine growth retardation; HTA: arterial hypertension; T2DM type 2 *diabetes mellitus*

As mentioned before, an interesting finding of studies that analyze the differential methylation in 45,X, 46,XX, and 47,XXX cells is that methylation-based and expression-based pathway analyses are complementary rather than overlapping. These findings could be due to the different methodologies employed, cell-tissue tested, and specific epigenetic memory (for example, hypomethylation of enhancers active during embryonic development, but dormant in adult tissues [96, 97]). Collectively, the findings in all of these studies suggest (1) a unique expression and DNA methylation profile for 45,X cell lines. Consequently, widespread differences in methylation and changes in global gene expression distinguish both TS (45,X) and control (46,XX and 46,XY) subjects. It should be noted that the most differently expressed genes were not located on the retained X chromosome but rather on the autosomes. (2) Sex chromosomal escape genes, X-Y homologous gene pairs, and pseudoautosomal genes are prone to differential expression in 45,X subjects. (3) A relationship between differential gene expression (and increased differential methylation) and comorbidities exists in TS. This differential gene expression includes (a) genes located on the autosomes and X chromosomes; (b) genes associated to autoimmune diseases; (c) genes associated to a distinct neuro-cognitive profile; (d) genes associated to urogenital congenital malformations; (e) genes associated to re-establishment pluripotency and germ cells (consequently associated with premature ovarian failure); (f) genes associated to obesity and disorders in metabolism, including T2DM and the metabolic syndrome; (g) genes associated to fetal development and embryonic lethality; (h) genes associated to sensorineural hearing loss; and (i) genes associated to aneuploidies. One should also stress that genes involved in the processes of epigenetic regulation may show a different DNA-methylation state in 45,X and 46,XX cells [13, 19]. This is consistent with the observation mentioned above of the role the absence of the second sex chromosome can cause on the deregulation of epigenetic modifications (Fig. 1).

A striking trait of TS is the wide clinical variability exhibited by non-mosaic 45,X individuals. However, intrauterine growth retardation (IUGR) and postnatal short stature are almost invariable characteristics of the X chromosome monosomy. These particular features have been associated with dyslipidemia and T2DM in adult non-TS subjects. This association is explained by an epigenetic adaptive mechanism called fetal reprogramming. Similarly, the IUGR seen in TS fetuses is also related to dyslipidemia and T2DM in adult TS patients, presenting with a higher incidence than that of the general population [98, 99]. Therefore, this epigenetic adaptation could partially compensate for the haploinsufficiency of the second sex chromosome in 45,X subjects, who may exhibit a constellation of clinical findings (in number and severity) depending on the specific epigenomes of each 45,X individual. Thus, the epigenome of each cell in 45,X subjects may be the result of haploinsufficiency of the second sex chromosome genes, and this in turn can have an influence over the effect of the haploinsufficient proteins on cells, organs, and tissues. To study this hypothesis, DNA of leukocytes from a phenotypically well-characterized cohort of adult patients with 45,X monosomy and age-matched female controls were tested. A total of 10,687 DMRs were identified on chromosomes 1, 9, 11, 17, 19 and 22 by linear models adjusting for the estimated relative cell proportions and age (sex chromosomes were excluded). To assign biological context, functional annotation clustering was performed using DAVID, which allowed for the identification of pathways involved in the development of congenital heart anomalies and coronary heart diseases, as well as for enrichment clusters of blood vessel development and transforming growth factor β. These pathways may account for the increased prevalence of congenital heart anomalies and increased incidence of aortic dissection seen in TS [13].

Transcription microarray-based studies have found that mRNA levels of some X chromosome-linked genes parallel the decrease in gene copy number in 45,X cells. However, studies of gene expression in 45,X versus 46,XX ESCs found evidence of under-expression in only a small number of genes on the X chromosome [14, 15, 17]. Conversely, some other X chromosome-linked genes lacked detectable decreases in expression in XO compared with euploid cells [40]. These differences can in some cases be explained by gene–gene interactions, when more than one gene in the same transcriptional pathway is present on the same X chromosome. Similarly, the fact that some genes on the X chromosome are not differentially expressed in monosomic cells can depend on the cell and tissue type. Finally, all gene expression studies have shown that X chromosome monosomic cells have many quantitative changes in gene expression, which is consistent with monosomy of sex chromosome leading to disturbances of transcriptional networks. Additionally, apart from simple transcriptional effects, a hypothesis that may explain TS pathogenesis, supported by an increasing number of studies that involve epigenetics, is that the presence of X chromosome monosomy could act in *trans* to produce tissue-specific modifications in DNA methylation and chromatin landscape at loci distributed throughout the genome [13]. These can then be inherited mitotically to daughter cells and change and/or hinder determined modifications in gene expression in developing tissues (Fig. 1).

## Contrast of epigenetic modifications in sex chromosome aneuploidies

Both TS and Klinefelter syndrome are associated with several clinical features that show a broad range of inter-individual expressivity and whose molecular mechanisms are poorly understood. Comprehending the effect of both the loss (45,X) and the gain (47,XXY) of the X chromosome on gene transcription and its correlation to their variable clinical phenotypes would be very helpful. Thus, systematic studies comparing genome-wide expression as well as epigenetic modifications of sex chromosome aneuploidies, e.g., 45,X versus 47,XXY, could help us gain insight into the mechanisms involved in their pathophysiology. Several studies have been conducted focusing only on one or another condition. In addition, such studies have analyzed a limited number of samples from a single available tissue (schizophrenic brain [100], testis [101], whole blood [84, 91, 102, 103], fibroblast [15, 19], or amniotic fluid [92]). To date, this issue has been addressed in both populations only by Sharma et al. [17]. This study demonstrated that loss of an X chromosome has a much more profound consequence than gain of an additional X chromosome, as about five times more loci are affected in TS than in KS subjects. In addition, when only autosomal CpG sites were analyzed, approximately 80% of the modifications in TS individuals represented hypomethylation of these loci, whereas nearly equal numbers of hypo- and hypermethylated CpG sites were observed in KS individuals [17]. Therefore, gene ontology analysis and the etiopathogenesis in both conditions may be completely different.

Several general conclusions can be reached by analyzing the results of the published articles on gene expression and epigenetic changes in the aneuploidies of sex chromosomes. First, gene ontology analysis reveals different clusters of genes involved in the clinical features of both TS and KS. Second, numerical abnormalities of the sex chromosomes affect not only the status of methylation in X-linked loci but also, and mainly, large numbers of autosomal loci. Third, in samples of TS subjects, the affected loci are characterized by intermediate methylation levels in 46,XY males/46,XX females. Nevertheless, samples from TS subjects are still closer to female than to male samples. Lastly, samples of KS individuals are significantly less methylated than samples of 46,XX subjects on strongly silenced loci on the X chromosome, which indicates a non-optimal X chromosome inactivation process in KS individuals. Although, in autosomal loci, samples from TS individuals are distinctly different from those exhibited by 46,XX and 46,XY subjects, samples from subjects with chromosomal constitutions 46,XX, 46,XY, and 47,XXY display a more or less similar methylation pattern. However, the second X chromosome inactivation in KS seems to be less operative than in 46,XX subjects. This opens the possibility that the specific DNA methylation-based biomarkers identified in these studies may largely distinguish samples of TS and KS subjects from female and male samples, respectively. Furthermore, these biomarkers can also be used for detailed molecular analysis of differential patterns of DNA methylation and epigenetic marks associated with sex chromosome aneuploidies.

## Haploinsufficiency and its epigenetic consequences in 45,X monosomy

Although the role of epigenetics in TS is currently far from understood, an increasing number of studies have demonstrated the key role of DNA methylation and gene expression on the etiopathogenesis of TS. As summarized in Table 1, various decontrolled (under/overexpressed) X chromosome-linked gene products are epigenetic modulators, thereby deregulating epigenetic mechanisms in TS. In turn, these disturbed mechanisms might contribute to the observed features and associated complications in TS.

**Table 1:** Altered gene expression due to X chromosome monosomy

Product	Primary function	Results in
DEAD (AspGlu-Ala-Asp) box helicase 3(DDX3)X/Y gene family	Involved in embryogenesis, spermatogenesis, cellular growth and division	No change in gene expression 45,X cells (17)
PRKX/Y	A protein kinase related to macrophage and granulocyte maturation.	No change in gene expression 45,X cells (17)
BMP2 (bone morphogenetic protein 2)	Bone metabolism	Downregulated (< 6-fold) in 45,X cells (17)
BMPER (BMP binding endothelial regulator)	A secreted protein that interacts with and inhibits BMPs function (bone metabolism)	Upregulated (> 5-fold) in 45,X cells (17)
SFRP1 (secreted frizzled-related protein 1)	Associated with Wnt signaling. Altered gene expression is associated with diminished osteoblast proliferation and differentiation	Downregulated (< 4-fold) in 45,X cells (17)
IGF-2 (insulin-like growth factor 2)	Related to regulation of carbohydrate metabolic process	Downregulated (< 22-fold) in 45,X cells (17)
ENPP1 (ectonucleotide pyrophosphatase/phosphodiesterase 1)	Related to regulation of carbohydrate metabolic process	Downregulated (< 22-fold) in 45,X cells (17)
TRIB3 (tribbles pseudokinase 3)	Involved in insulin signaling	Upregulated (> 4-fold) in 45,X cells (17)
SLC2A14 (solute carrier family 2 member 14 (facilitated glucose transporter))	Glucose transporter (GLUT)	Upregulated (> 18-fold) in the 45,X cells (17)
SLC25A6 (solute carrier family 25 member 6)	A gated pore that translocates ADP from the cytoplasm into the mitochondrial matrix and ATP from the mitochondrial matrix into the cytoplasm. Permeability transition pore complex (PTPC): regulates the release of mitochondrial products that induce apoptosis.	Downregulated (> 2-fold) in the 45,X cells (14)
CLDN11 (claudin 11)	Involved in spermatogenesis	Upregulated (> 18-fold) in the 45,X cells (17)
STC1 (stanniocalcin 1)	Paracrine regulation of follicular development	Downregulated (< 14-fold) in the 45,X cells (17)
RASD2 (RASD family, member 2)	Interfere with the functional activity of TSHR, FSHR, and LHCGR	Downregulated (< 5-fold) in the 45,X cells (17)
PRKX (protein kinase, X-linked)	A serine threonine protein kinase similar to the catalytic subunit of cyclic AMP dependent protein kinases. Involved in renal epithelial morphogenesis and in macrophage and granulocyte maturation. Deletion causes sex reversal disorder.	Downregulated (> 2-fold) in the 45,X cells (14) (11)
LANCL3 (LanC lantibiotic synthetase component C-like 3)	Signal transduction (inferred from biological aspects of ancestor)	Upregulated (> 1.67-fold) in 45,X cells (11)
RPS4X (ribosomal protein S4, X-linked)	A component of the 40S subunit	Downregulated (< 0.63-fold) in the 45,X cells (11)
ALAS2 (5′-aminolevulinate synthase 2)	An erythroid-specific mitochondrially located enzyme. It catalyzes the first step in the heme biosynthetic pathway.	Upregulated (> 2.19-fold) in 45,X cells (11)
GPR34 (G protein-coupled receptor 34)	An integral membrane protein. It mediate signals to the interior of the cell via activation of heterotrimeric G proteins that in turn activate various effector proteins, ultimately resulting in a physiologic response	Downregulated (< 0.59-fold) in the 45,X cells (11)
JPX transcript (XIST activator)	A nonprotein-coding RNA transcribed from a gene within the X-inactivation center. It participates in X chromosome inactivation by activating XIST on the inactive X chromosome	Downregulated (< 0.76-fold) in the 45,X cells (11)
EIF1AX (eukaryotic translation initiation factor 1A, X-linked)	An essential eukaryotic translation initiation factor. It is required for the binding of the 43S complex to the 5′ end of capped RNA	No change in gene expression 45,X cells (11)
ZFX (zinc finger protein, X-linked)	A transcriptional regulator for self-renewal of both stem cell types	No change in gene expression 45,X cells (11)
KDM6A (lysine demethylase 6A)	A tetratricopeptide repeat (TPR) protein. It catalyzes the demethylation of tri/dimethylated histone H3. Importance for reestablishment of pluripotency and germ cell development	Differentially methylated positions (11)
KDM5C (lysine demethylase 5C)	Involved in the regulation of transcription and chromatin remodeling	Differentially methylated positions (11)
USP9X (ubiquitin-specific peptidase 9, X-linked)	A protein similar to ubiquitin-specific proteases. Mutations may cause changes in the neuronal migration and axonal growth, resulting in intellectual disability	Differentially methylated positions (11)
UBA1 (ubiquitin-like modifier activating enzyme 1)	It catalyzes the first step in ubiquitin conjugation to mark cellular proteins for degradation. It also may function in DNA repair.	Differentially methylated positions (11)
STS (steroid sulfatase)	A multi-pass membrane protein that is localized to the endoplasmic reticulum and hydrolyzes several 3-beta-hydroxysteroid sulfates, which serve as metabolic precursors for estrogens, androgens, and cholesterol. Mutations in this gene are associated with X-linked ichthyosis (XLI	Differentially methylated positions (11)

## *O*-GlcNAcylation: a pathway contributing to epigenetic actions in 45,X monosomy

A number of studies have exposed several autosomal genes which are differentially expressed between XX and XO in human and mice models, revealing transcriptional changes due to monosomy of the X chromosome. In the mouse model, the findings obtained by microarray and qPCR were not fully concordant, illustrating the difficulty in ascertaining modest fold changes, such as those expected for genes escaping X chromosome inactivation. Remarkably, considerable variation was observed between tissues suggesting that inactivation patterns may be tissue-dependent [80, 94]. However, transcriptional analyses of XX, XY, and XO mice have uncovered a number of genes that exhibit both sex differences in gene expression and deregulation with X monosomy, such as the *OGT* gene, leading to genome-wide disturbances [80]. This gene encodes *O*-linked *N*-acetylglucosamine transferase (OGT), which adds *O*-GlcNAc onto serine and threonine residues of proteins. The importance of *O*-GlcNAcylation as critical posttranslational modifications for a wide array of cellular processes has been previously highlighted [104–106]. Interestingly, OGT is located on the X chromosome near the Xist locus (in humans at Xq13.1 and in mice at XqD). This location suggests that OGT is subject to the control of dosage compensation mechanisms in females [107, 108] so that its refined control is necessary for normal development and that its deregulation can contribute to an increased risk for a number of associated conditions in TS [104]. This hypothesis is supported by its involvement in disorders including cancer [109], neurodegeneration [110], cardiovascular disease [111], and T2DM [112]. This role for OGT on health could be due to the fact that *O*-GlcNAcylation has been identified on a large pool of intracellular proteins that have wide-ranging roles, including several epigenetic actions such as the complex formation with regulators of DNA demethylation, transcriptional repressors, transcriptional activator, histone methyltransferase, and polycomb repressive complex 2 [105]. Moreover, OGT is able to modify histones themselves [113] and to alter the C-terminal domain (CTD) of RNA polymerase II [114]. The epigenetic role of *O*-GlcNAcylation regulate complex processes such as cell cycle progression, cell signaling, and embryonic development [115], among others. Thus, OGT has emerged as a highly regulated nutrient-sensing epigenetic modifier that could modulate diverse expression networks, and its potential deregulation may contribute to the variable clinical features observed in TS patients.

Therefore, an abnormal expression of *OGT* in maternal or paternal retained X chromosomes might represent a link that explains the high lethality observed in 45,X conceptuses and the early-life origin of adult disease. Consequently, the increased risk of embryonic lethality and for common diseases of adult life could be a consequence of an OGT escape from the X chromosome inactive in the placenta. However, the parental origin of the retained single X chromosome does not appear to influence fetal survival [116], and the factor(s) causing the great lethality in human 45,X pregnancies do not seem to be X-imprinted.

Female rodents, who have experienced malnutrition during perinatal development, have an increased incidence of dyslipidemia [117]. Patients with TS present with low birth weight/length [98, 118, 119] and subsequently with an increased risk for weight gain [87], metabolic syndrome [120], impaired glucose intolerance and T2DM [84, 121, 122], and hypertension and ischemic heart disease [84, 87, 119, 122]. As *O*-GlcNAcylation is involved in the development of T2DM [112], it would not be unusual to imagine an involvement of OGT in the pathogenesis of these disorders in TS. An increased risk for T2DM among adult patients with TS, especially those with an isochromosome Xq has been observed [84]. A proposed explanation for this increased risk could be that haploinsufficiency for unknown Xp gene(s) constitutes a “first hit” that causes the basic deficit in pancreatic β-cell function seen in 45,X patients. Excess dosage of Xq genes in isochromosome Xq may provide a “second hit” that exacerbates the deficit, perhaps by altering other genes involved in pancreatic β-cell development and function or survival, and/or by stimulating low-grade chronic autoimmunity that injures, but does not obliterate the β-cells [84].

Interestingly, the human *OGT* gene is located on the Xq13 near the Xist locus, so that TS subjects with isochromosomes Xq have two OGT alleles on the same X chromosome, and thus, they could potentially overexpress *OGT*. In support of this hypothesis, an increased risk for T2DM has been observed among patients with Klinefelter syndrome (47,XXY) and 48,XXYY who have supernumerary copies of *OGT*, even in the absence of X-monosomy [123–125]. However, no study has been published so far in TS patients with an isochromosome Xq in which OGT levels have been tested and further research is needed to determine if this could be the cause for the increased incidence of T2DM in these patients. Similarly, although all observations suggest a relationship between OGT and the TS phenotype, particularly relating to cognitive function, visceral adiposity, and carbohydrate and lipid profiles, it still remains unclear whether OGT is deregulated in TS individuals. Consequently, further examination of OGT expression in these individuals would be of interest.

## Alterations of the immune response in Turner syndrome

Another key player gene on the X chromosome involved in epigenetic regulation is the UTX (ubiquitously transcribed tetratricopeptide repeat on X chromosome) gene. Turner syndrome is also associated with alterations of the immune response. T cell immune alterations and their relationship to haploinsufficiency of the *UTX* gene in the context of epigenetic regulation have been reported in TS subjects [91]. A gene expression microarray analysis performed on peripheral blood mononuclear cells (PBMCs) was carried out to identify potential pseudoautosomal X-linked genes that contribute to immune alterations in TS and to determine whether the gene expression in immune cells was altered in TS subjects. A total of 1169 unique genes showed differential expression between TS and control female PBMCs, including 35 genes on the X chromosome. *UTX* or *KDM6A* located at Xp11.3 [126] was found to be among the top 10 X chromosome linked genes with the largest decrease in expression [127]. Interestingly, *UTX* is the only gene among these candidates that escapes X inactivation [128] and is a histone H3 lysine 27 (H3K27) demethylase that epigenetically regulates gene expression. In addition, mice with T cell-specific deletion of *UTX* have an increased H3K27 methylation and a decreased expression at Il6ra and other Tfh (T follicular helper)-related genetic loci [127]. T cell-specific UTX deficiency knockout mice also have impaired clearance of chronic viral infection due to decreased frequencies of Tfh cells, which are critical for antibody generation by B cells. Thus, UTX is required for optimal CD4+ T cell differentiation to Tfh cells during chronic, but not acute, viral infection [127]. In parallel, reduced numbers of circulating CD4 CXCR5+ T cells (a measurable substitute of antibody production from Tfh cells) have been demonstrated in TS subjects with a decreased UTX expression in immune cells [127] compared to female controls. Thus, decreased *UTX* expression in TS subjects might increase their predisposition to viral infections due to Tfh cell deficiency with subsequent reduced antibody levels. Although all of this data suggests that *UTX* haploinsufficiency in TS individual immune cells has functional consequences, it is unclear whether TS patients also have an increased predisposition to chronic infection, for example, chronic otitis media, due to decreased Tfh cell numbers. In any case, the findings of decreased expression of UTX in TS immune cells, as well as the mechanism by which UTX affects CD4+ T cell differentiation to Tfh cells, are important steps toward understanding immune alterations in TS.

## Embryonic lethality

There is a well-described increase in intrauterine lethality in 45,X conceptuses [129]. The formation of 45,X embryos occurs very commonly during human reproduction, but subsequently, 99% of these embryos are spontaneously miscarried [130, 131]. Thus, 45,X is considered to be the most frequent genetic abnormality in the human species as it is present in approximately 2% of all conceptions that survive long enough to be clinically recognized pregnancies and about 15% of spontaneous abortions are 45,X. Therefore, early lethality is the most prevalent phenotype of the 45,X karyotype. In contrast to humans, XO mice are anatomically normal, fertile, and viable with no prenatal lethality [81]. Consequently, human TS cells or mouse models cannot be used to study the causes of early lethality in 45,X embryos. An alternative model to study the embryonic lethality is human XX ES cell lines, as these cells can differentiate in vitro into cells from the three embryonic germ layers, as well as into extra-embryonic cells. As mentioned above, a well-known feature of XX ES cell lines is the frequent loss of one of the two X chromosomes [33, 34, 40].

The high lethality of 45,X conceptuses occurs especially during the implantation period [129], which suggests the involvement of rather general etiopathogenic mechanisms [132]. If the 45,X embryos break through a certain barrier, then these global etiopathogenic mechanisms would provoke several accumulated related fetal or congenital defects. Three non-excluding hypothesis can be postulated to explain the high frequency of early lethality in 45,X embryos. First, in both morula and blastocyst stages, the pluripotent 45,X cells could be unstable, and therefore, differentiation is triggered too quickly and/or randomly. In this scenario, one would expect that the reprogramming of 45,X cells to iPSCs is not able to occur, since pluripotent 45,X cells would automatically degenerate or not transform into ESCs. However, using either skin fibroblasts or amniocytes from 45,X subjects, 45,X iPSCs have been generated [18], able to stably maintain their chromosomal constitution and with phenotypes similar to ESCs. In addition, these 45,X iPSCs were used for further assays including global gene expression analysis and tissue-specific directed differentiation [18].

The second hypothesis postulates that 45,X ESCs undergo a differentiation blockade, and consequently, certain tissue and/or structures cannot be formed properly leading to embryonic loss. In this case, 45,X embryos would either not truly differentiate into one or more of the three germ cell layers (ectoderm, endoderm, or mesoderm) or tolerate specific defects in certain cell lineages (e.g., cardiac mesoderm). However, 45,X iPSCs become rather complex teratomas and could produce, upon directed differentiation in vitro, specific lineages morphologically and functionally indistinguishable from those produced from ESCs or euploid iPSCs.

Thus, although the existence of subtle lineage-specific differentiation defects causing multi-organ failure and embryonic loss in TS fetuses is possible, a third scenario seems to be more feasible: inadequate global placentation and/or unbalanced cell proliferation/differentiation may disturb coordinated embryonic and extraembryonic tissue growth, eventually causing the embryos death. The findings of a reduced gene expression in X chromosome-linked genes in 45,X iPSCs support the existence of a different and more general etiopathogenic mechanism [18, 40].

A differential expression profile of genes that escape X inactivation was analyzed in a microarray analysis (Affymetyrix) from human XX ES cell lines [40]. Total RNA was extracted from populations of undifferentiated, in vitro and in vivo differentiated cells derived from human normal and XO ES cell lines. Out of 37 analyzed genes that escape X inactivation, 21 genes were expressed in differentiated and undifferentiated human XO ES cell lines. From these 21 identified genes, only three ARSE, STS, and TBL1X fulfilled the criteria of being a pseudoautosomal gene and to be expressed in 46,XX cells, but not expressed (or expressed at a very low level) in 45,X ES cells. None of these three candidate genes showed monoallelic expression, which implies that genomic imprinting does not play a major role in the early lethality of 45,X embryos. Gene expression microarray data was divided into lists of genes enriched in different tissues, and their gene expression of 46,XX and 45,X were compared in differentiated ES cell lines. The only tissue where many genes were expressed at higher levels in XX cells as compared to XO cells, both in vivo and after in vitro differentiation, was the placenta. All placental genes were expressed at least 5-fold higher in XX ES cells as compared to 45,X ES cell lines. Of these genes, *STS* and *CSF2RA* are X chromosome linked genes that escape X chromosome inactivation and were found to be highly enriched in the placenta. However, the *STS* gene has no active homolog on the Y chromosome [133], and therefore, it is not likely that haploinsufficiency of this gene causes the abnormal placental differentiation. *CSF2RA* (colony-stimulating factor 2 receptor alpha) gene encodes the alpha subunit of the receptor of granulocyte-macrophage colony-stimulating factor (CSF2 or GMCSF) which is an essential autosomal gene for normal placental development [134]. It is remarkable that *CSF2RA* is expressed 9.5-fold more in 46,XX cells than in 45,X cells [40]. It is therefore possible that haploinsufficiency of *CSF2RA* may lead to abnormal placental differentiation. This, in turn, may cause an epigenetical downregulation of many placental genes, including *CSF2RA* itself, which might explain its higher differential expression level in 46,XX than in 45,X ES cell lines. It is also intriguing to see that *Csf2ra* in mice is an autosomal gene. This might explain the less severe phenotype seen in XO mice versus that seen in 45,X embryos, a hypothesis previously explored [81]. This also suggests that the genes involved in 45,X early lethality phenotype are regulated epigenetically by a pseudoautosomal X chromosome linked gene. Overall, these data point out that at least one of the reasons for the early lethality of 45,X embryos is abnormal placental differentiation as a result of haploinsufficiency of X-linked pseudoautosomal genes. The effect of X monosomy on placental differentiation may occur very early on and may therefore have a general influence on various components of the placenta.

In addition, 45,X iPSCs have been also used for studies of global gene expression analysis and tissue-specific directed differentiation [18]. Lower levels of gene expression were found for the pseudoautosomal gene *PPP2R3B* in multiple clones when compared with euploid cell controls. These 45,X cell clones could be transformed into neural-like, hepatocyte-like, and heart-like cells. *PPP2R3B* is a pseudoautosomal gene whose product is a subunit of protein phosphatase 2A which acts as a negative regulator of cell proliferation and is important for the correct exit of mitosis during early embryonic cell division [135, 136]. Tentatively, the haploinsufficiency of the *PPP2R3B* gene in 45,X conceptuses might alter cell proliferation during early embryonic development and indirectly also affect tissue-specific differentiation due to improper synchronization. Although, no increased proliferation in 45,X iPSCs when compared with the controls was shown [18], it is possible that the mitotic defect is more relevant in vivo and/or becomes apparent when 45,X blastocysts differentiate. In addition, these clones displayed insufficient upregulation of CSF2RA during embryoid body formation [18]. Taken together, the findings described in 45,X iPSCs support the notion that abnormal organogenesis and early lethality in 45,X conceptuses are not caused by a tissue-specific differentiation blockade but may rather involve other abnormalities including impaired placentation.

On the other hand, the essential role of OGT for embryonic and extraembryonic tissue development has been demonstrated in the knockout model. Ogt knockout mouse embryos die in the blastocyst period [106], suggesting that OGT is required during pre-implantation development. Ogt has been identified as having decreased expression in XO mouse placenta [94] and has not been shown to undergo genomic imprinting on the inactivated X chromosome in XX extra-embryonic tissue [137]. Consequently, *Ogt* expression is biallelic in XX mouse trophobast stem cells, as XX placentas have higher levels of gene expression of Ogt and higher concentrations of *O*-GlcNAcylated proteins than XY placentas [138]. Accordingly, the embryonic lethality observed in mice that inherited a maternal mutant X results from decreased Ogt expression during pre-implantation development, rather than from decreased expression in extraembryonic tissues [139]. However, taking into consideration the fetal reprogramming hypothesis, the differential dosage of *O*-GlcNAcylation, and by extension OGT differential expression between 45,X and 46,XX placentas, may play a role in processes that contribute to parent-of-origin effects such as gluconeogenesis, lipogenesis, and cardiac metabolism [111].

## Future implication of epigenetic therapies on clinical features and associated complications in Turner syndrome

Due to the probable impact of epigenetic changes on the TS phenotype, resulting from X chromosome monosomy, the potential role of epigenetic treatment for the care and management of TS should be considered. First, hypomethylating agents such as 5-azacytidine, while useful as anticancer epidrugs, are mutagenic and, therefore, not appropriate for use in other clinical conditions such as TS [11, 19]. Treatment approaches based on dietary management can also affect DNA methylation patterns, and they are known to be nontoxic and safe and may, thus, be used as potential therapeutic agents in preventing or improving the associated metabolic complications frequently seen in adult patients with TS. The classical example of a methyl donor is the supplementation with folic acid and vitamin B12. Although not expected to reduce methylation on hypermethylated loci, it might prevent losses of methylation at hypomethylated loci, therefore helping to maintain methylation levels genome-wide. However, to our knowledge, no studies have so far examined this type of therapy in individuals with TS in controlled trials. This therapeutic approach, based on dietary manipulation, has been demonstrated to have variable and partly promising effects on measures of overall health in other disorders [140]. On the other hand, identification of target master regulator genes that can play key roles in the development of clinical features and associated complications, such as immune deregulation and short stature, may provide clues to future therapies, both in TS and in other conditions. Lastly, the most promising of the therapy-based modifications of gene expression is the technique of epigenetic editing in which specific epigenetic enzymes, such as a DNMT or HAT, are recruited to specific genes by means of a lab-engineered DNA-binding domain. Consequently, epigenetic changes are actively superscript, leading to potentially continuing modulation of gene expression. Plausibly, the expression of under-expressed X chromosome-linked genes might produce physiological levels that might improve short stature and impaired glucose and lipid metabolism, among other complications of TS.

## Summary and future perspectives

Even though the etiology of TS (monosomy of the second sex chromosome) has been known for many decades, how this aneuploidy leads to the TS phenotype is still not well understood. Clinicians and scientists believed that TS was a contiguous gene syndrome resulting from deleted genes carried on the second sex chromosome which gave rise to short stature, gonadal dysgenesis, typical visible dismorphic stigmata, and associated urinary, cardiovascular, skeletal, and endocrine abnormalities. So far, the prevailing hypothesis, based on rare structurally abnormal X and Y chromosome case reports, supports gene dosage effects, in which decreased expression of X chromosome-linked genes lead to direct altered biological outcomes and interrupts and/or modifies many downstream transcriptional pathways. Thus, it is widely assumed that the TS phenotype results from dosage imbalance of the genes located on the second sex chromosome (X or Y chromosome). Although the deletion of genes on the second sex chromosome would theoretically lead to a 0.5-fold decrease in the gene expression level, the mRNA levels of many X chromosome-linked genes deviate from this. Whereas most researchers have tried to identify these genes and its contribution to TS features, the underlying cause of this gene expression variation has been largely ignored. In this respect, epigenetic mechanisms are of crucial importance in gene expression and thus might play a central role in the development of the TS phenotype. DNA methylation and mRNA transcription studies suggest a disruption of pathways involving the “skeletal system,” “gonadal development and function,” “glucose metabolism,” and “epigenetic regulation” and that various genetic mechanisms may contribute to the clinical features of TS and its phenotypic variability.

Studies on the expression of mRNA in cells and tissues with X chromosome monosomy have shown that, while some genes on the X chromosome are under-expressed, other subsets of genes on other chromosomes show an altered expression. This could be due to a variety of mechanisms including the activity of transcription factors encoded on the X chromosome or elsewhere in the genome that are affected by this aneuploidy, leading to alterations in DNA methylation, posttranslational histone modifications, nucleosomal core assembly, and chromatin remodeling through miRNAs and lncRNAs. Recent results support the synthetic view to explain the etiopathogenesis of TS. The analyses of the epigenome of TS subjects have demonstrated DMRs that, although enriched on the X chromosome, are also involved in most of the chromosomes. Significantly, several of these DMRs were functionally correlated with pathways pathologically linked to developmental defects in TS. Surprisingly, epigenetic mechanisms have been poorly evaluated in TS. Most TS studies have focused on genetic aspects, ignoring the current evidence that points toward the contribution of epigenetics to the TS phenotype. Therefore, in this review, we have tried to summarize and to assess the limited information available on the effect of epigenetics in the etiopathogenesis of TS. We have also offered further evidence for the role of epigenetics on short stature, embryonic lethality, cardiac defects, impaired glucose, and lipid metabolism that have been extracted from methylation and gene expression studies, including those of TS patients. As TS probably results from gene expression disturbances, research of the molecular mechanisms that regulate phenotypic expression require an understanding of the transcriptome differences present in 45,X monosomy cells and tissues, which have been previously explored by several studies. However, the extensive natural gene expression variation occurring in both 45,X and 46,XX cells complicates the identification of changes related to 45,X monosomy per se. In this review, we proposed that the under-expression of several genes modifies the chromatin landscape of the nuclear compartment in 45,X monosomy cells. These modifications would lead to a general disturbance of the transcriptome that might explain some of the clinical features seen in TS. Since constitutive global hypomethylation and specific hypermethylated loci in TS affect multiple tissues, including blood, it may provide useful biomarkers for TS, as well as pharmacological targets for therapeutic intervention. Significantly, recent epigenetic therapies (epidrugs and epigenetic editing) are already being used for cancer and epilepsy and might offer new, different, and innovative possibilities for the treatment of short stature and for the common adult complications seen in TS patients. To our knowledge, no studies have so far explored epigenetic therapy in TS. However, it may provide potentially important novel pathways in the care and management of TS patients.

An interesting focus for future research may be the epigenetic involvement of the GH-IGF-I axis in the etiopathogenesis of short stature, hearing loss, and common diseases of adult life in TS. A hypothesis proposed in 1990 by Barker is that IUGR has a causal relationship in the origins of hypertension, coronary heart disease, and DMT2 in adulthood, so that a stimulus or insult at a critical, sensitive period of early life has permanent effects on structure, physiology, and metabolism later in life (fetal programming) [141, 142]. Fetal programming may result from adaptations that occur when the maternal–placental nutrient supply fails to match the fetal nutrient demand. Intrauterine growth retardation is a constant clinical feature of X chromosome monosomy [98, 118]. Multiple factors can condition the presence of IUGR in TS [7]. In general, IUGR decreases serum insulin growth factor-1 (IGF-1) levels, and a reduced mRNA IGF1 expression has been demonstrated in TS fibroblasts, which suggests a reduced autocrine/paracrine action of IGF-1 in TS [143]. Additionally, reduced free IGF-I and increased IGFBP-3 proteolysis in TS, modulated by female sex steroids, has been described in women with TS [144]. IGF1 is an epigenetically regulated gene that has two promoters, alternative exon 5 splicing, and multiple termination sites [145, 146]. Conservation nucleotides and amino acids of IGF-I in human and rat species, as well as a comparable expression of multiple mRNA variants, have been noted for both species [147]. Also, a profile of epigenetic signatures in the GH-IGF-1 axis has been described in IUGR model rats (a recent review examined this topic [148]). Due to the evolutionarily conserved and biochemical and genetic similarities of the GH-IGF-I axis in both species, this pattern of epigenetic marks in the murine models might be extrapolated to the human. The early developmental epigenetic maturation pattern may be essential for the maintenance of an optimal GH-IGF-I axis during infancy, childhood, adolescence, and adult life in TS, and it can be altered by IUGR. Also, mRNA IGF1 variants might be used as markers of altered transcriptional regulation and may function to monitor the growth and response to rhGH in TS subjects. In addition, a study has shown that hearing loss in TS was related to serum concentrations of IGF-1 and height [149]. Also, altered lipid and glucose profiles were related with a low birth weight and length in TS [98]. Thus, IUGR, and its impact on epigenetic signatures during fetal life, may play a role in the onset, course, associated complications, and therapy of common diseases of adult life in this group of patients. Therefore, TS might be considered another example of fetal programming and the consequences of it. For example, although epigenetic mechanisms can affect the regulation of several gene pathways throughout life and explain the gene–environment interaction, more importantly, they are potentially modifiable and consequently reversible. Growth hormone (rhGH) therapy at supraphysiological doses is known to improve the final height of TS subjects. Knowing the regulatory epigenetic pattern of the human *IGF1* gene would help identify an accessible chromatin around this locus in TS patients. This may lead to the use of a lower dose of rhGH in TS subjects with fewer adverse side effects, opening the possibility of a more personalized medicine.

Another interesting and remarkable mechanism, not explored until now, but which might gain acceptance in the next years, it is our understanding of the epigenetic regulation of transcriptional control of metabolic diseases of adult life in TS. Metabolic syndrome (MetS) is a cluster of common disorders that include visceral adiposity, hypertension, insulin resistance, and dyslipidemia. This disorder is associated with an increased risk for cardiovascular disease, myocardial infarction, stroke, and T2DM. The frequency of each component of MetS in adult TS patients is high. The atherosclerotic process starts early [55, 90, 121, 150, 151] in TS, and epidemiological data suggests that there is an associated 3-fold increase in the risk of mortality from cardiovascular and cerebrovascular diseases, when compared to that of the general female population [119]. Thus, the life expectancy in TS is reduced by at least 10 years [90, 119]. An increased risk of insulin resistance, hypertension, glucose intolerance, dyslipidemia, and liver dysfunction have been associated with adult TS patients [84, 90, 150, 151]. Insulin resistance and dyslipidemia are probably the most common metabolic abnormalities in normal-weight adult patients with TS. Insulin resistance and dyslipidemia are frequently related to the disorders in apolipoproteins, receptors, enzymes, or cofactors in proteins related to glucose and/or lipoprotein metabolism [152]. Peroxisome proliferator-activated receptors (PPARs) are members of the second class of nuclear receptors [152]. PPARs act through genomic, as well as nongenomic mechanisms and their activity can be modified posttranslationally [153] and regulated by epigenetic mechanisms. Among them, PPARγ is involved in adipocyte differentiation, lipid and glucose metabolism, mitochondrial biogenesis, and regulation of inflammatory pathways [154]. Exploring the role of epigenetic regulation, as a potential modifier of the metabolic phenotype seen in TS, may be of interest in future research. In particular, analyzing the epigenetic aspects of genes such as peroxisome proliferative-activated receptor c coactivator 1 alpha (*PPARGC1A*) might open new routes of research, due to altered signaling of this gene which have been described as contributing to glucose intolerance, insulin resistance and T2DM and control of mitochondrial biogenesis.

Thus, there is now a growing consciousness in the clinical field that having the correct pattern of epigenetic signatures is critical for the development of a normal phenotype. If epigenetic mark patterns in the GH-IGF1 axis are not properly established or maintained during the fetal period, as may occur in TS, conditions as diverse as short stature, hearing loss, hypertension, T2DM, dyslipidemia, and cardiovascular disease may appear. However, much more research will be required before translating these findings into clinical practice.

## Conclusions

In conclusion, the phenotypic consequences of epigenetic modifications in TS are being evaluated. These studies will require assessment of the epigenetic roles of upstream effector genes on the X chromosome, including DNA methylation- and histone modification-pathway genes, transcriptional genes and non-codifying RNA (miRNA), and the groups of downstream target genes that are affected epigenetically by the X chromosome monosomy.

## Abbreviations

APC/C: Anaphase-promoting complex/cyclosome
CSF2RA: Colony-stimulating factor 2 receptor alpha gene
DAVID: Database for Annotation, Visualization and Integrated Discovery
DMRs: Differentially methylated regions
DNMT: DNA methyltransferase
DNMT3B: DNA methyltransferase 3b gene
DSBs: Double-strand breaks
ESCs: Embryonic stem cells
H3K27: Methylation of lysine 27 residues at histone 3
H3K9me2: Histone H3 lysine 9 di-methylation
HAT: Histone acetyltransferase
ICF: Immunodeficiency, Centromeric instability and Facial anomalies
IUGR: Intrauterine growth retardation
lncRNAs: Noncoding large RNA molecules
MI, MII: Meiosis I and II, respectively
miRNA: Noncoding microRNA molecules
mRNA: Messenger RNA
MSCI: Meiotic sex chromosome inactivation
OGT: O-linked *N*-acetylglucosamine transferase
PBMCs: Peripheral blood mononuclear cells
SHOX: Short stature homeobox-containing gene
T2DM: Type 2 diabetes mellitus
Tfh: T follicular helper
TS: Turner syndrome
UTX: Ubiquitously transcribed tetratricopeptide repeat on X chromosome gene
